# The Association of IFN-γ, TNF-α, and Interleukins in Bronchoalveolar Lavage Fluid with Lung Cancer: A Prospective Analysis

**DOI:** 10.3390/jpm13060968

**Published:** 2023-06-08

**Authors:** Patricia Hogea, Emanuela Tudorache, Ovidiu Fira-Mladinescu, Monica Marc, Diana Manolescu, Felix Bratosin, Ovidiu Rosca, Adelina Mavrea, Cristian Oancea

**Affiliations:** 1 Center for Research and Innovation in Precision Medicine of Respiratory Diseases, “Victor Babes” University of Medicine and Pharmacy, Eftimie Murgu Square 2, 300041 Timisoara, Romania; hogea.patricia@umft.ro (P.H.); tudorache_emanuela@yahoo.com (E.T.); mladinescu@umft.ro (O.F.-M.); marc.monica@umft.ro (M.M.); dmanolescu@umft.ro (D.M.); oancea@umft.ro (C.O.); 2 Doctoral School, Faculty of General Medicine, “Victor Babes” University of Medicine and Pharmacy, Eftimie Murgu Square 2, 300041 Timisoara, Romania; 3 First Pulmonology Clinic, Clinical Hospital of Infectious Diseases and Pulmonology, “Victor Babes”, Gheorghe Adam Street 13, 300310 Timisoara, Romania; 4 Second Pulmonology Clinic, Clinical Hospital of Infectious Diseases and Pulmonology, “Victor Babes”, Gheorghe Adam Street 13, 300310 Timisoara, Romania; 5 Discipline of Radiology, “Victor Babes” University of Medicine and Pharmacy Timisoara, Eftimie Murgu Square 2, 300041 Timisoara, Romania; 6 Discipline of Infectious Diseases, “Victor Babes” University of Medicine and Pharmacy Timisoara, Eftimie Murgu Square 2, 300041 Timisoara, Romania; felix.bratosin@umft.ro (F.B.); ovidiu.rosca@umft.ro (O.R.); 7 Department of Internal Medicine I, Cardiology Clinic, “Victor Babes” University of Medicine and Pharmacy Timisoara, Eftimie Murgu Square 2, 300041 Timisoara, Romania

**Keywords:** lung cancer, cytokines, bronchial lavage, diagnosis

## Abstract

Lung cancer is a leading cause of cancer-related mortality worldwide. Identifying novel diagnostic and prognostic biomarkers is essential for improving patient outcomes. This study aimed to investigate the predictive role of cytokines from bronchoalveolar lavage fluid (BALF) in lung cancer diagnosis and prognosis. A prospective study was conducted on 33 patients with suspected lung cancer, divided into inflammatory and non-inflammatory BALF groups. Inflammatory markers in BALF were assessed, and their association with lung cancer risk was analyzed using receiver operating characteristic (ROC) plot analysis, sensitivity and specificity percentages, and regression analysis. Statistically significant differences were observed between the inflammatory and non-inflammatory groups for several inflammatory markers, including IFN-gamma, IL-1b, IL-2, IL-6, IL-10, and IL-12p70. In the follow-up analysis, significant differences persisted for IFN-gamma, IL-1b, IL-2, IL-4, and IL-6. ROC plot analysis revealed that IL-12p70 had the highest area under the curve (AUC) value (0.702), followed by IL-2 (0.682), IL-6 (0.620), IL-4 (0.611), TNF-alpha (0.609), IL-10 (0.604), IL-1b (0.635), and IFN-gamma (0.521). IL-6 showed the highest sensitivity (73%), and IL-1b had the highest specificity (69%). Regression analysis demonstrated that IL-6 (cut-off = 25 pg/mL) and IL-12p70 (cut-off = 30 pg/mL) had the highest odds ratios for lung cancer risk, at 5.09 (95% CI: 2.38–9.24, *p* < 0.001) and 4.31 (95% CI: 1.85–8.16, *p* < 0.001), respectively. Cytokines from BALF, particularly IL-6 and IL-12p70, show potential as diagnostic and prognostic biomarkers for lung cancer. Further studies with larger cohorts are warranted to confirm these findings and elucidate the clinical implications of these markers in lung cancer management.

## 1. Introduction

Lung cancer continues to be a major public health concern, with an increasing number of people being diagnosed and succumbing to the disease worldwide. As the leading cause of cancer-related deaths in both men and women, it is imperative to develop strategies for early detection, diagnosis, and treatment [1,2,3,4]. Despite advancements in therapeutic approaches, the 5-year survival rate for lung cancer patients remains low, at approximately 15% [5,6]. This poor prognosis can be attributed to the fact that most patients are diagnosed at advanced stages when treatment options are limited [7,8,9]. The lack of noninvasive clinical tests for early diagnosis and screening further exacerbates this issue. Thus, there is an urgent need to identify specific biomarkers for accurate and timely lung cancer diagnosis [10].

Chronic inflammation has been recognized as a critical factor in carcinogenesis, contributing to various stages of cancer development, including malignant transformation, invasion, and metastasis [11,12]. Both innate and adaptive immune responses illustrate the functional link between inflammation and cancer. Inflammatory and tumor cells secrete cytokines and chemokines, which are proteins that modulate cellular and humoral system activity [13,14]. Understanding the role of cytokines in the context of lung cancer can provide valuable insights for identifying novel diagnostic and prognostic markers and aid in developing targeted therapies.

Current research efforts focus on elucidating the role of cytokines in lung cancer and identifying potential biomarkers that can be used for diagnosis, prognosis assessment, and therapy response evaluation [15]. Several studies have demonstrated the potential utility of detecting specific cytokines, such as interleukins, tumor necrosis factors, and tumor growth factors, in bronchoalveolar lavage fluid or blood samples for the differential diagnosis of lung cancer and proliferation of cancer cells [16]. Inflammatory cytokines such as IFN-gamma, IL-1b, IL-2, IL-4, IL-6, IL-10, IL-12p70, and TNF-alpha have been shown to be involved in the immune response associated with cancer progression [17]. IFN-gamma, primarily produced by NK cells and T cells, plays a vital role in enhancing antigen presentation and the cytotoxic activity of T cells. IL-1b, a pro-inflammatory cytokine, is known to promote angiogenesis and invasiveness of tumor cells. Similarly, IL-2 is a cytokine secreted by T cells in response to antigen stimulation and is crucial for T cell proliferation and NK cell activity. Other interleukins may promote tumor growth by inhibiting effector T-cell function and enhancing regulatory T-cell function [18]. 

As such, it is hypothesized that the analysis of these selected cytokines in BALF could provide comprehensive insight into the inflammatory and immune responses in lung cancer, offering potential diagnostic and prognostic value. Thus, the present study aims to investigate the diagnostic and prognostic value of cytokines derived from bronchoalveolar lavage fluid in patients with lung cancer. Through the identification of reliable biomarkers, this research seeks to contribute to the development of more accurate diagnostic methods and improve the clinical management of lung cancer patients, ultimately leading to better patient outcomes.

## 2. Materials and Methods

### 2.1. Study Design

A prospective study was performed to measure the inflammatory markers in the bronchoalveolar fluid lavage of patients with bronchopulmonary cancer and determine their diagnostic and prognostic utility. All of the invasive procedures and scopes of the current study were explained to the patients before inclusion, and informed consent was obtained from all subjects willing to participate. This research was conducted according to the guidelines of the Declaration of Helsinki and approved by the Institutional Review Board of the Hospital of Infectious Diseases and Pulmonology “Victor Babes”, from Timisoara, Romania, on 23 September 2022, with the number 10218. 

### 2.2. Study Cohort

Patients admitted to the Pneumology Clinic of the “Victor Babes” Clinical Hospital from Timisoara between September 2022 and February 2023 were included in this study. All of the patients were required to match all of the following inclusion criteria: (1) patients with a major suspicion of bronchopulmonary cancer on the chest computed tomography (CT), (2) the need to perform bronchoscopy for diagnostic purposes, (3) patients with a Karnofsky performance status ≥60% [19], and (4) those who were confirmed with a diagnosis of lung cancer by histological analysis after bronchoscopy were included in the current study. 

On the other side, the exclusion criteria of all participants were: (1) severe heart failure NYHA III and IV [20], (2) contraindications for bronchoscopy [21], (3) the patient’s refusal to participate in the study, and (4) patients without endoscopic characteristics of bronchopulmonary cancer [22]. All of the patients and controls underwent bronchoscopy for diagnostic purposes before the initiation of treatment. All of the bronchoscopies were performed by one researcher, based on the hospital guidelines, in the same hospital unit. At the end of the study period, 33 patients were diagnosed with lung cancer and were further split into two study groups based on their BALF inflammatory status. One group comprised lung cancer patients with inflammatory cytology that had a high neutrophil density and an overall high density of inflammatory cells [23]. On the contrary, the remaining patients with lung cancer and non-inflammatory BALF cytology were allocated to a separate group. Patients that underwent bronchoscopy but were not diagnosed with lung cancer were considered the control group. The control group included patients with diffuse interstitial pneumopathy (hypersensitivity pneumonitis, sarcoidosis, nonspecific interstitial pneumonia—NSIP), obstructive pulmonary pathologies and patients with a chronic cough that had a suspicion for malignancy and required a diagnostic bronchoscopy. All of the patients underwent bronchoscopy twice, initially for diagnosis and measurement of BALF cytokine profile and second time for follow-up of BALF cytokine levels.

The variables considered for inclusion and analysis comprised the following: age, age range, body mass index (BMI), BMI proportions, smoking status, pack-year number, exposure to respiratory hazards, the Modified Medical Research Council (mMRC) scale of dyspnea [24], distance from first symptom onset, Charlson Comorbidity Index, spirometry measurements (functional expiratory volume), diagnostic studies (position of the tumor, presence of metastasis, tumor size, lung biopsy findings, immunohistochemistry), and BALF analysis comprising: IFN-gamma, IL-1b, IL-2, IL-4, IL-6, IL-10, IL-12p70, and TNF-a.

### 2.3. Laboratory Analysis

All of the participants underwent flexible bronchoscopy using Olympus bronchoscopes under either moderate sedation with propofol or general anesthesia, based on the clinician’s judgement. The bronchoalveolar lavage process involved the injection of 200 mL of normal saline in four 50 mL portions into the bronchus leading to the most significant radiological lesion. BALF was collected with gentle suction into a plastic cylinder. Approximately 50 mL was set aside for regular clinical tests, BALF was collected, centrifuged, and the supernatant was stored for future analysis. The study selected certain cytokines verified in earlier research as significant factors in the tumor microenvironment of lung cancer, each of these cytokines playing distinct roles, acting as markers for certain cell activities, promoting or inhibiting carcinogenesis, angiogenesis, immune evasion, or apoptosis, and influencing prognosis and response to treatments.

The selected cytokines were analyzed by employing a multiplex array on a Luminex platform, using a total of 40 high-sensitivity kits. This comprehensive array incorporated a broad range of cytokines, including the ones previously mentioned. The acceptance criteria were established, which included variables such as coefficient of variation, lower limit of detection, and linearity. These procedures were designed to ensure accurate, comprehensive, and meaningful analysis of the collected specimens.

### 2.4. Statistical Analysis

GraphPad Prism version 6.0 for Microsoft Windows, was used to conduct the statistical analysis (GraphPad Software U.S.A.). The Kolmogorov–Smirnov test was used to assess the normality of the data. The mean value, which represents central tendency, and the standard deviation, which measures dispersion, were used to represent normally distributed data. Student’s *t*-test was used to examine the difference in means between the two comparison groups of normally distributed data, while the Mann–Whitney u-test was used to compare non-Gaussian variables. To compare the mean differences between the three groups, the ANOVA test was employed. The Chi-square test was used to compare proportions between the two study groups, while Fisher’s exact test was used in case the frequency assumption was not fulfilled. A receiver operating curve (ROC) and area under the curve (AUC) were used to plot the accuracy of the BALF cytokines for lung cancer diagnosis, and the Youden index was used to determine the optimal threshold for the identified markers. The AUC values, which range from 0 to 1, reflect the ability of each inflammatory marker to discriminate between patients at risk for lung cancer and those not at risk. The closer the AUC value is to 1, the better the marker’s discriminative ability. A *p*-value below 0.05 was regarded as being statistically significant. 

## 3. Results

### 3.1. Patients’ Background Characteristics

A total of 33 patients were enrolled in the current study. Table 1 presents the background data of the study participants, which were divided into two groups: those with inflammatory bronchoalveolar lavage fluid (*n* = 22) and those with non-inflammatory BALF (*n* = 11). The mean age of participants in the inflammatory group was 60.3 years, and 59.2 years in the non-inflammatory group. The mean BMI was 23.2 kg/m^2^ for the inflammatory group and 22.3 kg/m^2^ for the non-inflammatory group, with no significant difference between the two groups (*p* = 0.588). 

Gender distribution was also analyzed, with 68.2% of participants in the inflammatory group being male, compared to 54.5% in the non-inflammatory group (*p* = 0.442). In terms of smoking history, 59.1% of the inflammatory group were smokers or ex-smokers, compared to 63.6% of the non-inflammatory group, with no significant difference between the two groups (*p* = 0.801). The average pack-year smoking was approximately 30 in both groups. The mMRC dyspnea score of 3–4 was observed in 22.7% of the inflammatory group and 36.4% of the non-inflammatory group, with no significant difference (*p* = 0.407). The CCI score of >2 was observed in 72.7% of the inflammatory group and 45.5% of the non-inflammatory group. In terms of the degree of respiratory dysfunction (FEV1), no significant difference was observed between the two groups (*p* = 0.601). 

Table 2 describes the diagnostic studies performed on the two groups of patients. The position of the tumor was central in 54.5% of the inflammatory group and 63.6% of the non-inflammatory group (*p* = 0.618). Metastasis was present in 59.1% of the inflammatory group and 18.2% of the non-inflammatory group, with a statistically significant difference between the groups (*p* = 0.026). Regarding tumor size, no significant difference was observed between the two groups (*p* = 0.314), most patients in the inflammatory group had tumors between 5 and 7 cm in a proportion of 35.4%, while 54.5% had tumors between 3 and 5 cm in the non-inflammatory group.

No significant difference was found between the two groups in terms of lung biopsy findings (*p* = 0.969). In the inflammatory group, the majority of patients had an SCLC histology (40.9%), while in the non-inflammatory group, 54.5% had ACC. Immunohistochemistry results were reported for fifteen patients in the inflammatory group and eight patients in the non-inflammatory group. The markers examined included programmed cell death ligand 1 (PD-L1), anaplastic lymphoma receptor tyrosine kinase gene (ALK), and epidermal growth factor receptor (EGFR). No significant difference was observed between the two groups in terms of the immunohistochemistry results (*p* = 0.908). 

### 3.2. BALF Analysis

**Figure 1:**
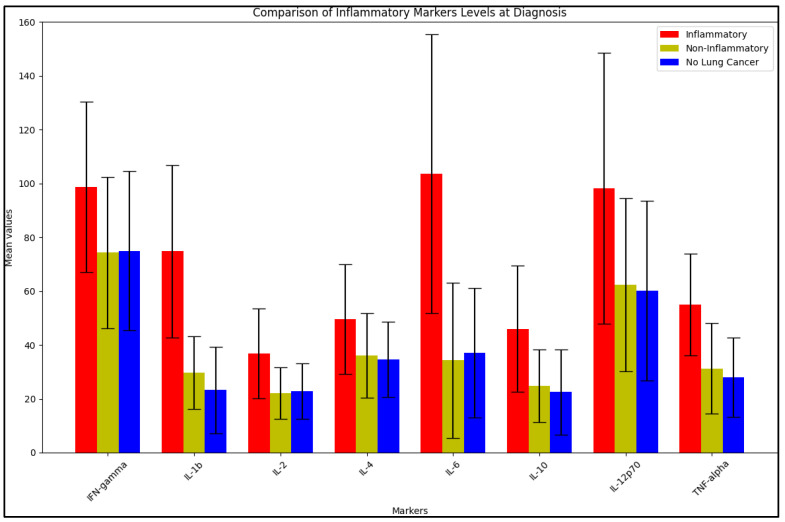
BALF cytokines at diagnosis.

The data in Table 3 describe the BALF analysis of inflammatory markers in lung cancer patients with inflammatory BALF, non-inflammatory BALF, and the control group, respectively. At diagnosis, the analysis revealed statistically significant differences between the comparison groups for several inflammatory markers. IFN-gamma levels were significantly higher in the inflammatory group (98.7 ± 31.6 pg/mL) than in the non-inflammatory group (74.3 ± 28.1 pg/mL) with a *p*-value of 0.038. IL-1b levels were also significantly higher in the inflammatory group (74.8 ± 32.1 pg/mL) compared to the non-inflammatory group (29.7 ± 13.5 pg/mL) with a *p*-value of <0.001. Similar significant differences were observed for IL-2 (*p* = 0.010), IL-6 (*p* < 0.001), IL-10 (*p* = 0.009), and IL-12p70 (*p* = 0.040). TNF-alpha levels were also significantly higher in the inflammatory group (55.1 ± 18.9 pg/mL) compared to the non-inflammatory group (31.3 ± 16.7 pg/mL) with a *p*-value of 0.001, as seen in Figure 1.

**Figure 2:**
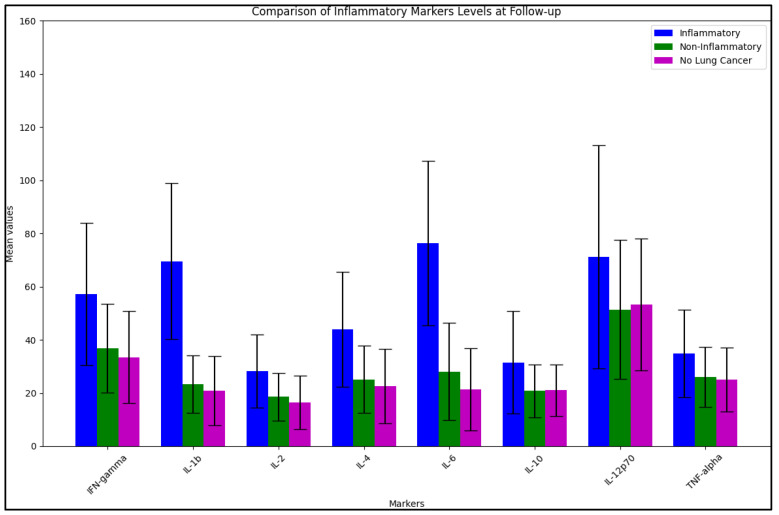
BALF cytokines at follow-up.

In the follow-up analysis, significant differences between the inflammatory and non-inflammatory groups persisted for several markers. IFN-gamma levels remained significantly higher in the inflammatory group (57.2 ± 26.8 pg/mL) than in the non-inflammatory group (36.9 ± 16.7 pg/mL) with a *p*-value of 0.038. IL-1b levels continued to be significantly higher in the inflammatory group (69.6 ± 29.3 pg/mL) compared to the non-inflammatory group (23.3 ± 10.9 pg/mL) with a *p*-value of <0.001, as observed in Figure 2. Similar significant differences were observed for IL-2 (*p* = 0.044), IL-4 (*p* = 0.011), and IL-6 (*p* < 0.001). 

**Figure 3:**
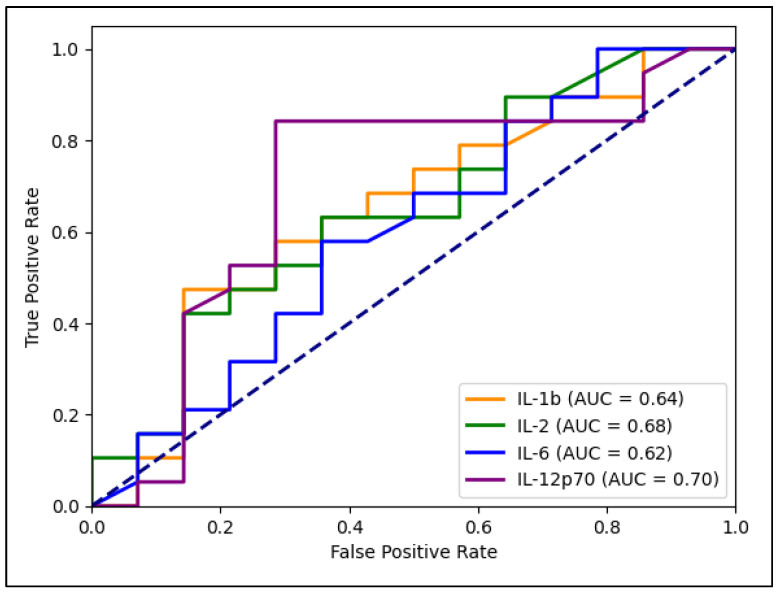
ROC plot analysis.

Table 4 presents the receiver operating characteristic (ROC) plot analysis for the inflammatory markers involved in estimating the risk for lung cancer. IL-12p70 showed the highest AUC value (0.702), followed by IL-2 (0.682), IL-6 (0.620), IL-4 (0.611), TNF-alpha (0.609), IL-10 (0.604), IL-1b (0.635), and IFN-gamma (0.521). Statistical significance was observed for IL-1b (*p* = 0.044), IL-2 (*p* = 0.040), IL-6 (*p* = 0.001), IL-10 (*p* = 0.008), IL-12p70 (*p* = 0.001), and TNF-alpha (*p* = 0.046), indicating that these inflammatory markers are significantly associated with the risk of lung cancer. The sensitivity and specificity percentages reflect the marker’s ability to correctly identify patients at risk for lung cancer (sensitivity) and those not at risk (specificity). IL-6 showed the highest sensitivity (73%), followed by IL-12p70 (70%). In terms of specificity, IL-1b had the highest specificity (69%), followed by IL-12p70, as described in Figure 3.

Table 5 presents the regression analysis of the adjusted factors for the inflammatory markers. IL-6 (2nd generation) showed the highest odds ratio of 5.09 (95% CI: 2.38–9.24, *p* < 0.001), implying that patients with IL-6 levels above 25 pg/mL have a 5.09 times higher risk of lung cancer compared to those with lower levels. IL-12p70 also showed a significantly increased risk with an odds ratio of 4.31 (95% CI: 1.85–8.16, *p* < 0.001) for patients with levels above 30 pg/mL.

IL-2 and IL-1b demonstrated significant associations with lung cancer risk as well, with odds ratios of 2.16 (95% CI: 1.20–4.91, *p* = 0.009) and 1.88 (95% CI: 1.09–3.54, *p* = 0.030), respectively, for patients with levels above 17 pg/mL and 22 pg/mL. IL-10 showed a modest association with lung cancer risk, with an odds ratio of 1.30 (95% CI: 0.98–2.02, *p* = 0.066) for patients with levels above 13 pg/mL. However, this association was not statistically significant. Lastly, TNF-alpha did not significantly affect lung cancer risk, with an odds ratio of 1.19 (95% CI: 0.94–2.38, *p* = 0.118) for patients with levels above 34 pg/mL.

## 4. Discussion

### 4.1. Literature Analysis

In the current study, the only statistically significant difference between the inflammatory and non-inflammatory groups was the presence of metastasis. No significant differences were found between the groups regarding tumor position, tumor size, lung biopsy findings, or immunohistochemistry results. Additionally, the BALF analysis revealed significant differences between patients with inflammatory and non-inflammatory BALF in several inflammatory markers at both diagnosis and follow-up. These findings suggest that these markers may play a role in differentiating between inflammatory and non-inflammatory lung conditions. Moreover, the ROC plot analysis revealed that several inflammatory markers, including IL-1b, IL-2, IL-6, IL-10, IL-12p70, and TNF-alpha, are significantly associated with the risk of lung cancer. These markers demonstrated varying degrees of sensitivity and specificity in estimating lung cancer risk. The results suggest that these inflammatory markers may have potential value as diagnostic tools for assessing lung cancer risk.

In this study, we chose cytokines previously established as significant factors in the lung cancer tumor microenvironment. As such, TNF-α, an alveolar macrophage activity marker, contributes to carcinogenesis inhibition. IL-1b and IL-6 are also markers for alveolar macrophage activity and can influence lung cancer survival prognosis [17]. IL-2 strongly activates natural killer cells, and IL-4 shows higher expressions in NSCLC. IL-8 attracts immune cells and induces angiogenesis, and high levels indicate reduced survival in lung cancer. IL-10 is immunosuppressive and associated with tumor progression. IL-12 and IL-12p70 activate natural killer cells and alveolar macrophages, while IL-13, which was not evaluated in the current study, signifies natural killer cell activity and is linked to lung cancer progression and metastasis. Other interleukins, such as IL-17, promote angiogenesis and cell proliferation and inhibit apoptosis, correlating with tumor progression. Finally, IL-23 hampers the activity of B, T, and natural killer cells, promoting lung cancer progression and metastasis [18]. However, the latter two were not measured in patients’ BALF in this study. In addition to the already tested markers in the current study, CRP is an acute-phase protein produced in the liver in response to the high level of cytokines secondary to an inflammatory stimulus. Additionally, CRP is the most commonly studied marker of chronic inflammation [25,26]. Several studies have shown that circulating levels of CRP were increased in patients with different types of cancer, such as lung, liver, breast, pancreatic, ovarian, and stomach cancer [27,28]. Several possible mechanisms have been proposed to explain the relationship between CRP and cancer: chronic lung inflammation can have an etiological role in the development of cancer by creating a tissue microenvironment that could cause potentially malignant DNA changes; tumor growth can induce tissue inflammation and secondary increase in CRP levels; however, the CRP levels can also be increased due to other causes such as smoking or acute inflammation [29].

TNF-α is an inflammatory cytokine produced by activated macrophages, tumor cells, and inflammatory cells in the tumor microenvironment. This cytokine is involved in several processes related to cancer, such as immune response, inflammation, growth, cell proliferation and differentiation, apoptosis, and metastasis [30]. Similarly, IL-10 is a multifunctional cytokine with immunosuppressive and anti-angiogenic functions. According to studies, IL-10 has both pro-tumorigenic activity and activity in tumor inhibition through these functions. The opposite effects of IL-10 probably depend on its interactions with other cytokines or with various factors in the tumor microenvironment [31].

Several studies have shown that patients with lung cancer have a higher serum level of CRP than those with benign lung pathologies or healthy people [32,33], and those with a high level of CRP have a high risk of developing lung cancer. Additionally, the prognosis of patients with lung cancer and elevated CRP level is poorer [34,35]. Other research shows that the level of serum ferritin is significantly higher in patients with lung cancer compared to patients with benign lung pathologies, although this was not tested in the current study [36,37]. Zhou et al. observed higher levels of serum ferritin and CRP in patients with lung cancer, and regarding the non-small cell lung cancer (NSCLC) subtype, they measured higher values of these parameters in patients with adenocarcinoma compared to those with squamous cell carcinoma [38]. Additionally, a meta-analysis showed that increased serum ferritin concentration was significantly associated with worse overall survival [39]. Some studies have shown elevated levels of ferritin in other types of cancer than lung cancer. Thus, these results indicate that serum ferritin could be a common phenomenon in cancer patients and that it could be a useful tumor marker [40,41].

Another study, which was the largest of its kind, evaluated local and systemic concentrations of different cytokines in lung cancer patients before and during radiotherapy (RT) [42]. The results demonstrated that serum and bronchoalveolar lavage fluid IL-6 and serum IL-8 were higher in lung cancer patients compared to non-cancer controls, suggesting that lung cancer itself upregulates the production of IL-6 and IL-8 and that the action of RT further increases BALF IL-6. It was also noted that BAL fluid IL-6 was further elevated during radiotherapy, consistent with a previous observation that BALF TGF-b1 and IL-6 levels rise during RT for lung cancer [43]. The increased IL-6 levels during RT could be attributed to a local inflammatory reaction, a response by the immune system to RT, or the release of IL-6 due to the destruction of tumor cells. Additionally, the authors observed a tendency for a vascular endothelial growth factor (VEGF), IL-8, and IL-18 in BALF to rise during RT, a finding not previously reported. In vitro studies have demonstrated that there is considerable individual variation in the secretion of interleukins, which may explain why the only significant interleukins increase during RT was noted for IL-6 [44,45].

Furthermore, another study found that higher baseline serum and BALF IL-8 and serum VEGF levels were associated with reduced survival of lung cancer patients, primarily those with squamous cell carcinoma [46]. This finding is consistent with previous research that has reported high tumor levels of IL-8 mRNA, VEGF mRNA, and protein, as well as positive staining of tumor-cell VEGF-C and VEGFR-3, to be associated with significantly shorter survival of lung cancer patients [47]. Notably, this study was the first to establish that IL-8 in BALF might be a prognostic factor for overall survival. These findings contribute to the growing body of evidence supporting the predictive role of cytokines from bronchoalveolar lavage fluid in lung cancer diagnosis and prognosis, and they emphasize the importance of continued research to better understand the underlying mechanisms and clinical implications of these biomarkers.

The potential discovery of cytokine biomarkers could play an essential role in disease management once the lung cancer diagnosis has been established, helping determine prognosis and guide treatment decisions. Additionally, if particular cytokine patterns are confirmed to be highly predictive in subsequent larger studies, there may be potential to develop non-invasive tests, such as breath or blood tests, based on these markers. Thus, even though our research involves an invasive procedure, the long-term goal is to contribute to the body of knowledge that could lead to the development of non-invasive diagnostic and prognostic tools for lung cancer.

### 4.2. Study Limitations

One primary limitation of the current study is the relatively small sample size, with only 33 patients included, which may limit the generalizability of the findings. Additionally, the study cohort was derived from a single center, which may introduce potential selection bias and limit the applicability of the results to broader populations. Another limitation is the lack of a healthy control group, as the comparison group included patients with non-inflammatory cytology. This may affect the interpretation of the diagnostic and prognostic utility of inflammatory markers in relation to a truly healthy population. Moreover, the study did not account for potential confounding factors such as the patient’s history of other inflammatory conditions, immunosuppressive medication use, or previous history of cancer, which could influence the levels of inflammatory markers.

Furthermore, all of the bronchoscopies were performed by a single researcher, which may have introduced potential biases related to the researcher’s technique and interpretation of the results. Finally, the study focused solely on the analysis of cytokines in BALF and did not consider the potential utility of other diagnostic methods or biomarkers that might provide a more comprehensive understanding of lung cancer diagnosis and prognosis. Future studies addressing these limitations, such as enrolling larger and more diverse patient cohorts, including a healthy control group, and investigating additional diagnostic and prognostic markers, would be beneficial to validate and expand upon the current findings. 

## 5. Conclusions

In conclusion, patients with inflammatory BALF cytology exhibited higher levels of specific inflammatory markers, including IFN-gamma, IL-1b, IL-2, IL-6, IL-10, and IL-12p70, compared to those with non-inflammatory BALF and the control group. The follow-up analysis showed persistently higher levels of some markers, such as IFN-gamma, IL-1b, IL-2, IL-4, and IL-6. The ROC plot analysis revealed that IL-1b, IL-2, IL-6, IL-10, IL-12p70, and TNF-alpha had a significant association with lung cancer, although with relatively low discriminative performance. Furthermore, the regression analysis demonstrated that patients with lung cancer had significantly higher odds for increased levels of IL-1b, IL-2, IL-6, and IL-12p70 above the calculated cut-off values. Further studies with larger sample sizes and longer follow-up periods are needed to investigate the implications of these inflammatory cytokines in BALF and explore the implications of utilizing these inflammatory markers in lung cancer diagnosis, prognosis, and management.

## Figures and Tables

**Table 1:** Background data of the study participants.

Variables	Inflammatory (*n* = 22)	Non-Inflammatory (*n* = 11)	*p*-Value
Age (mean ± SD)	60.3 ± 7.5	59.2 ± 8.4	0.705
Age range	34–75	41–84	
BMI (mean ± SD)	23.2 ± 4.7	22.3 ± 3.9	0.588
BMI categories			0.721
18.5–24.9 (kg/m^2^)	1 (4.5%)	1 (9.1%)	
25–29.9 (kg/m^2^)	13 (59.1%)	5 (45.5%)	
>30 (kg/m^2^)	8 (36.4%)	5 (45.5%)	
Gender (male, %)	15 (68.2%)	6 (54.5%)	0.442
Smoker/Ex-smoker (yes, %)	13 (59.1%)	7 (63.6%)	0.801
Pack-year smoking (mean ± SD)	31.5 (24.5–38.0)	33.0 (22.0–39.5)	0.548
Exposure to respiratory hazards (yes, %)	11 (50.0%)	3 (27.3%)	0.213
mMRC dyspnea (3–4)	5 (22.7%)	4 (36.4%)	0.407
Symptom onset, months (mean ± SD)	5.6 ± 3.7	5.8 ± 4.0	0.869
CCI > 2	16 (72.7%)	5 (45.5%)	0.124
Degree of respiratory dysfunction (FEV1)			0.601
Mild (≥80)	10 (45.5%)	3 (27.3%)	
Moderate (50–79)	9 (40.9%)	6 (54.5%)	
Severe (30–49)	3 (13.6%)	2 (18.2%)	

BMI, Body Mass Index; SD, Standard Deviation; mMRC, modified Medical Research Council; CCI, Charlson Comorbidity Index; FEV, Forced Expiratory Volume.

**Table 2:** Diagnostic studies.

Variables	Inflammatory (*n* = 22)	Non-Inflammatory (*n* = 11)	*p*-Value
Position of the tumor (central, %)	12 (54.5%)	7 (63.6%)	0.618
Metastasis (n, %)	13 (59.1%)	2 (18.2%)	0.026
Tumor size			0.314
≤3 cm	4 (18.2%)	3 (27.3%)	
3–5 cm	7 (31.8%)	6 (54.5%)	
5–7 cm	8 (36.4%)	2 (18.2%)	
>7 cm	3 (13.6%)	0 (0.0%)	
Lung biopsy findings			0.969
SCC	7 (31.8%)	3 (27.3%)	
ACC	6 (27.3%)	6 (54.5%)	
SCLC	9 (40.9%)	2 (18.2%)	
Immunohistochemistry	(*n* = 15)	(*n* = 8)	0.908
PD-L1	7 (46.7%)	4 (50.0%)	
ALK	3 (20.0%)	2 (25.0%)	
EGFR	5 (33.3%)	2 (25.0%)	

SCC, Squamous Cell Cancer; ACC, Adenocarcinoma; SCLC, Small Cell Lung Cancer; PD-L1, Programmed cell Death Ligand 1; ALK, Anaplastic Lymphoma Receptor Tyrosine Kinase Gene; EGFR, Epidermal Growth Factor Receptor.

**Table 3:** BALF analysis.

Inflammatory Markers	Normal Range *	Inflammatory (*n* = 22)	Non-Inflammatory (*n* = 11)	*p*-Value	No Lung Cancer (*n* = 7)	*p*-Value *
At diagnosis						
IFN-gamma (3rd gen.)	<2 pg/mL	98.7 ± 31.6	74.3 ± 28.1	0.038	75.0 ± 29.6	0.146
IL-1b	<12 pg/mL	74.8 ± 32.1	29.7 ± 13.5	<0.001	23.3 ± 16.1	<0.001
IL-2	<5 pg/mL	36.9 ± 16.7	22.1 ± 9.6	0.010	22.8 ± 10.4	0.007
IL-4	<5 pg/mL	49.5 ± 20.4	36.0 ± 15.7	0.063	34.6 ± 13.9	0.038
IL-6 (2nd gen.)	5–15 pg/mL	103.6 ± 51.8	34.3 ± 28.9	<0.001	37.1 ± 24.0	<0.001
IL-10	<5 pg/mL	46.0 ± 23.5	24.8 ± 13.4	0.009	22.5 ± 15.8	0.002
IL-12p70	<3 pg/mL	98.2 ± 50.3	62.4 ± 32.1	0.040	60.2 ± 33.4	0.016
TNF-alpha	<16 pg/mL	55.1 ± 18.9	31.3 ± 16.7	0.001	28.0 ± 14.7	0.005
Follow-up						
IFN-gamma (3rd gen.)	<2 pg/mL	57.2 ± 26.8	36.9 ± 16.7	0.038	33.5 ± 17.2	0.028
IL-1b	<12 pg/mL	69.6 ± 29.3	23.3 ± 10.9	<0.001	20.8 ± 13.0	<0.001
IL-2	<5 pg/mL	28.2 ± 13.7	18.6 ± 9.0	0.044	16.4 ± 10.1	0.114
IL-4	<5 pg/mL	44.0 ± 21.6	25.1 ± 12.6	0.011	22.6 ± 13.9	0.001
IL-6 (2nd gen.)	5–15 pg/mL	76.3 ± 30.9	28.0 ± 18.3	<0.001	21.3 ± 15.5	<0.001
IL-10	<5 pg/mL	31.5 ± 19.3	20.8 ± 9.9	0.095	21.0 ± 9.7	0.043
IL-12p70	<3 pg/mL	71.1 ± 42.0	51.4 ± 26.2	0.166	53.2 ± 24.8	0.015
TNF-alpha	<16 pg/mL	34.9 ± 16.5	26.0 ± 11.3	0.118	25.1 ± 12.0	0.219

* Group analysis and subgroup comparisons performed using the Analysis of Variance (ANOVA) test; IFN, Interferon; IL, Interleukin; TNF, Tumor Necrosis Factor.

**Table 4:** ROC plot for the inflammatory markers involved in estimating the risk for lung cancer.

Variables	AUC	95% CI		SE	%	%	*p*-Value
		Lower Bound	Upper Bound		Sensitivity	Specificity	
IFN-gamma (3rd gen.)	0.521	0.454	0.619	0.136	63%	45%	0.118
IL-1b	0.635	0.516	0.762	0.083	66%	69%	0.044
IL-2	0.682	0.531	0.816	0.070	69%	65%	0.040
IL-4	0.611	0.487	0.741	0.108	52%	66%	0.092
IL-6 (2nd gen.)	0.620	0.539	0.728	0.064	73%	69%	0.001
IL-10	0.604	0.524	0.730	0.077	65%	61%	0.008
IL-12p70	0.702	0.598	0.805	0.081	70%	66%	0.001
TNF-alpha	0.609	0.526	0.752	0.089	62%	64%	0.046

AUC, Area Under Curve; CI, Confidence Interval; SE, Standard Error; IL, Interleukin; IFN, Interferon; TNF, Tumor Necrosis Factor.

**Table 5:** Regression analysis.

Adjusted Factors	Cut-Off	Odds Ratio	(95% CI)	*p*-Value
IL-1b	22 pg/mL	1.88	1.09–3.54	0.030
IL-2	17 pg/mL	2.16	1.20–4.91	0.009
IL-6 (2nd gen.)	25 pg/mL	5.09	2.38–9.24	<0.001
IL-10	13 pg/mL	1.30	0.98–2.02	0.066
IL-12p70	30 pg/mL	4.31	1.85–8.16	<0.001
TNF-alpha	34 pg/mL	1.19	0.94–2.38	0.118

CI, Confidence Interval; IL, Interleukin; IFN, Interferon; TNF, Tumor Necrosis Factor.

## Data Availability

Data are available upon request.
